# Prevalence and Factors Associated with Potassium Abnormalities Among Outpatients with Heart Failure Taking Diuretics in a Tertiary Referral Hospital in Tanzania

**DOI:** 10.24248/eahrj.v8i1.762

**Published:** 2024-03-28

**Authors:** Hadija Bushahu, Bahati Wajanga, Benson Kidenya, Igembe Nkandala

**Affiliations:** a Morogoro Regional Referral Hospital, Morogoro, Tanzania; b Bugando Medical Centre, Mwanza, Tanzania; c Catholic University of Health and Allied Sciences, Mwanza, Tanzania

## Abstract

**Background:**

Heart failure (HF) is a chronic progressive condition in which the heart is unable to pump enough blood to meet the body's need for blood and oxygen. Globally, about 64 million people are affected with HF. This study was undertaken to determine the magnitude and factors associated with potassium abnormalities in heart failure patients on diuretics in Tanzania.

**Methods:**

This was a hospital based cross-sectional study conducted at Bugando Medical Centre's outpatient clinic. The selection of this hospital was driven by the significant presence of heart failure patients in the facility and the convenience for the researchers, who were stationed at this facility during the study period. All consenting adult patients aged 18 years and above that were attending the cardiac clinic and had met Framingham criteria for heart failure and were taking diuretics for at least one month were included.

**Results:**

The prevalence of hypokalemia and hyperkalemia was found to be 4.3% And 19.3% respectively. The median (IQR) age was 61 (46-70) years and majority of them (52.8%) were females. most of the patients (87.7%) had New York Heart Association (NYHA) class III heart failure.

**Conclusion:**

Factors associated with potassium abnormalities include medication use, kidney disease and more severe heart failure. Healthcare providers should ensure that all patients with these associated factors receive regular electrolyte testing. Electrolytes should be part of the baseline investigation to all patients with heart failure starting treatment, and should be closely monitored in every clinic visit for early detection of these abnormalities and possible intervention, including dose adjustments.

## BACKGROUND

Heart failure (HF) is a chronic progressive condition in which the heart is unable to pump enough blood to meet the body's need for blood and oxygen.^1^ Globally, about 64 million people are affected with HF. Despite the significant advances in therapies and prevention, the mortality and morbidity associated with HF are still high.^2^ Hypertension has been reported as the dominant cause of HF in Africa, responsible for up to 46% of cases of HF in hospitalised patients, cardiomyopathy account for 20 to 30% and ischemic heart disease for 7 to 9%.^2,3,4^

The prevalence of hypokalemia in the general population is 1 to 2% based on data from western countries, whereas the prevalence among patients with HF on diuretics has been reported to be 8.5% from the same countries.^5^ Hypokalemia is a marker of increased neurohormonal activity and disease progression and it may lead to ventricular arrhythmias and sudden death. Hypokalemia has been associated with increased rate of hospitalisation^6^ as well as mortality in patients with HF.^5,6^

Compared to the prevalence of hyperkalemia in the general population of 2%,^7^ the prevalence of hyperkalemia has been reported to be as high as 10%^8^ in patients with heart failure. Patients with HF are especially susceptible to hyperkalemia due to advanced age and comorbidities such as chronic kidney disease (CKD) that cause alteration in potassium excretion. In addition, the optimal medical treatment for HF involves the use of angiotensin converting enzyme inhibitors (ACEIs), angiotensin receptor blockers (ARBs), mineracortocoid receptor antagonists (MRAs) and betablockers that increase the risk of hyperkalemia. Hyperkalemia, if left untreated, may lead to a decrease in cardiac conduction, widening of the QRS complex and ventricular arrhythmia that can lead into asystole and sudden death.^9^

Although these abnormalities have been shown to be associated with increased morbidity and mortality, data regarding these abnormalities in Tanzania and Africa is lacking. This study was undertaken to determine the magnitude and factors associated with potassium abnormalities in heart failure patients on diuretics in Tanzania.

## METHODS

### Study Design and Settings

This was a hospital based cross-sectional study conducted at Bugando Medical Centre's outpatient clinic. Bugando Medical Centre is a referral hospital in the Lake zone of northern Tanzania with a catchment population of approximately 16 million. The selection of this hospital was driven by the significant presence of heart failure patients in the facility and the convenience for the researchers, who were stationed at this facility during the study period.

### Study Population

All consenting adult patients aged 18 years and above that were attending the cardiac clinic and had met Framingham criteria^10^ for heart failure and were taking diuretics for at least one month were included. Patients met the criteria if they had 2 major criteria or 1 major criterion with 2 minor criteria. The major criteria were; paroxysmal nocturnal dyspnoea, neck vein distention, rales and s3 gallop; the minor criteria were; bilateral lower limb edema, nocturnal cough, hepatomegaly and tachycardia.^10^ Patients with insufficient documented information to ascertain the diagnosis of heart failure were considered to have incomplete medical records and were excluded. Medical records were retrieved from the electronic system of the hospital as well as physical files from the hospital's records department. The sample size was calculated using the Leslie Kish formula^11^ with an estimated minimum number of patients of 138 assuming the prevalence of hyperkalemia of 10%^8^ with a margin of error of 5% and an alpha value of .05. (The sample size was also powered for detection of hypokalemia with an assumption of prevalence of 8.5%,^5^ with a margin of error of 5% and an alpha value of .05, which required a minimum sample of 120 subjects).

### Data Collection

A standardised pretested Swahili questionnaire and a data collection form were used to capture demographic and clinical information that included medical history and physical examination data. The questionnaire was pretested among clinic attendants with heart failure during the pilot phase of the study. Venopuncture was performed in all patients eligible for the study whereby 4 ml of blood was drawn and put into red-capped Vacutainer tubes that were immediately taken to the laboratory for serum potassium measurement using Cobas Integra ® 400 plus automated chemistry analyzer ((Integra; Roche, Switzerland). Hyperkalemia was defined as serum potassium of more than 5.5 mmol/L; levels between 5.5 mmol/L and 6 mmol/L were classified as mild hyperkalemia and between 6.1 mmol/L and 7 mmol/L moderate. On the other hand, hypokalemia was defined as serum potassium below 3.5 mmol/L whereby between 3.4 mmol/L and 3 mmol/L was classified as mild hypokalemia and between 2.5 mmol/L and 2.9 mmol/l moderate.^11,12^

### Data Processing and Analysis

Data from the questionnaire was entered into Microsoft Excel; analysis was performed using STATA version 13. Categorical variables were summarised as proportions. Continuous variables were summarised using medians and Interquartile Ranges (IQRs) or means and Standard Deviations (SDs). Factors associated with potassium abnormalities were determined using bivariate followed by multivariable logistic regression. A P value <.05 was considered statistically significant.

### Ethical Considerations

Confidentiality was maintained throughout the study and patients' participation in the study was voluntary; all participants provided an informed consent. Participants were free to decline from the study even after signing the consent at any time. Ethical clearance for the study was obtained from the Joint Catholic University of Health and Allied Sciences - Bugando Medical Centre (CUHAS-BMC) Research Ethics and Ethical committee with the research clearance certificate number CREC/311/2018. The laboratory results were immediately communicated with the attending physicians for appropriate interventions.

## RESULTS

### Baseline Social Demographic Characteristics

Between October 2018 and January 2019, a total of 305 patients with heart failure attending medical outpatient clinic were recruited into the study. The median (IQR) age was 61 (46-70) years and majority of them (52.8%) were females. Most of the patients (87.7%) had New York Heart Association (NYHA) Class III heart failure. Other characteristics are shown in Table 1.

**TABLE 1:** Baseline Social Demographic Characteristics (N = 305)

Variable	Frequency / median	Percentage / (IQR)
Age, years	61	[46 – 70]
Gender		
Male	144	47.2
Female	161	52.8
Marital status		
Single	21	6.9
Married	225	73.7
Widow(er)	59	19.3
Education level		
Informal	84	27.54
Primary	160	52.46
Secondary	40	13.11
College	21	6.9
Alcohol use		
No	229	75.1
Yes	75	24.6
Unknown	1	0.33
Duration of heart failure		
< 52 weeks	278	91
≥52 weeks	26	8.5
NYHA class		
II	87	28.7
III	178	87.5
IV	38	12.5
Occupation		
Employed	29	9.51
Unemployed	233	76.39
Retired	43	14.1

NYHA-New York Heart Association

### Clinical Characteristics of Study Participants

Most patients (58.7%) presented with muscle weakness, whereas 21% had nocturnal cough and 24.6% had paroxysmal nocturnal dyspnoea. On physical examination, majority (96.7%) had displaced apex beat and the median (IQR) systolic blood pressure (SBP) was 120 (110-135) mm Hg. Most patients (90.2%) were using beta blockers (Table 2).

**TABLE 2:** Clinical Characteristics of Study Participants (N = 305)

Variable	Frequency / median	Percentage/(IQR)
Blood pressure, mm Hg		
SBP	120	110–135
DBP	71	63–82
Current medications		
ACEi	251	82.2
ARB	52	17.05
Loop diuretics	297	97.37
Thiazide diuretics	12	3.9
Beta-blockers	275	90.16
MRA	59	19.34
NSAIDs	13	4.26
Metformin	15	4.92
Insulin	1	0.33
HIV status		
Yes	6	1.97
No	226	72.13
Unknown	79	25.9
Renal failure		
No	271	88.85
Yes	24	7.87
Unknown	10	3.28
Liver failure		
No	286	93.7
Yes	4	1.3
Unknown	15	4.92
Diabetes mellitus		
No	268	87.87
Yes	16	5.25
Unknown	21	6.9

SBP – Systolic Blood Pressure, DBP – Diastolic Blood Pressure, NSAIDs – Non-Steroidal Ant-Inflammatory Drugs, MRA – Mineralocorticoid Receptor Antagonist, ARB – Angiotensin Receptor Blockers, ACEi-Angiotensin Converting Enzyme Inhibitors, HIV – Human Immunodeficiency Virus

### Prevalence of Potassium Abnormalities

The prevalence of hypokalemia and hyperkalemia was found to be 4.3% and 19.3% respectively as indicated in Table 3.

**TABLE 3:** Distribution of Potassium Levels Among Study Participants (N = 305)

Electrolyte Abnormalities	Frequency (n)	Percentag (%)
Normal potassium	233	76.4
Hypokalemia	13	4.3
Mild hypokalemia (N=13)	12	92.3
Moderate hypokalemia (N=13)	1	7.7
Hyperkalemia	59	19.3
Mild hyperkalemia (N=59)	49	83.1
Moderate hyperkalemia (N=59)	10	16.9

### Factors Associated with Hypokalemia

Factors that were associated with hypokalemia were elevated systolic and diastolic blood pressure (BP) and usage of nonsteroidal anti-inflammatory drugs (NSAIDs) (Table 4).

**TABLE 4:** Factors Associated with Hypokalemia (N = 305)

Variables	OR (95% CI)	*P-Value*
Age (years)		
<50	1.0	
≥50	1.4 (0.37–5.25)	.302
Sex		
Female	1.0	
Male	0.51 (0.15–1.71)	.280
NYHA class		
II	1.0	
III	0.57 (0.18–1.83)	.344
IV	0.46 (0.05–3.97)	.478
Blood pressure (mm Hg)		
SBP	1.04 (1.01–1.07)	.008
DPB	1.05 (1.00–1.09)	.033
Use of NSAIDs		
No	1.0	
Yes	9.69 (2.18–43.13)	.003
Creatinine level	0.99 (0.97–1.01)	.490

Abbreviations: SBP – Systolic Blood Pressure, DBP – Diastolic Blood Pressure, NYHA-New York Heart Association, NSAIDs – NonSteroidal Ant-Inflammatory Drugs, CI, Confidence Interval, OR, Odds Ratio

### Factors Associated with Hyperkalemia

Hyperkalemia was more likely to be observed in patients with advanced heart failure, elevated BP, history of confusion, muscle weakness, vomiting and elevated creatinine on bivariate logistic regression analysis. However, on multivariable analysis, the only factors that were associated with hyperkalemia were serum creatinine level and SBP (Table 5).

**TABLE 5:** Factors Associated with Hyperkalemia (N = 305)

Variable	OR (95% CI)	P-Value	aOR (95% CI)	*P Value*
Age (years)				
<50	1.0			
≥50	1.35 (0.69–2.62)	.611		
Gender				
Female	1.0			
Male	1.37 (0.77–2.43)	.280		
NYHA class				
II	1.0			
III	3.42 (1.46–7.98)	.005		>.20
IV	3.92 (1.35–11.32)	.012		>.20
Confusion				
No	1.0			
Yes	5.09 (1.64–15.78)	.005	2.78 (0.78–9.89)	.114
Muscle weakness				
No	1.0			
Yes	2.4 (1.25–4.49)	.008	1.89 (0.95–3.75)	.067
Nausea				
No	1.0			
Yes	3.00 (1.09–8.25)	.033		>.20
Vomiting				
No	1.0			
Yes	5.57 (1.21–25.63)	.027		>.20
Blood pressure (mm Hg)				
SBP	1.03 (1.01–1.04)	.001	1.02 (1.007–1.04)	.006
DBP	1.02 (1.001–1.05)	.041		>.20
Creatinine level	1.01 (1.002–1.01)	<.001	1.004 (1.001–1.008)	.004

## DISCUSSION

In this study, we found a lower prevalence of hyperkalemia than the 25% reported by Reimar et al in Denmark.^14^ We believe the lower prevalence of hyperkalemia in our study, which had a median participant age of 61 years, can be attributed to the younger demographic compared to the Danish study where participants were older, with a median age of 78 years. Additionally, 41% of the subjects in the Danish study had chronic kidney disease, a known risk for hyperkalemia,^15^ in contrast to only 7.9% of patients in our study who had renal failure. Furthermore, Arampatzis et al.^16^ reported a hyperkalemia prevalence of 4% among patients on diuretics for various reasons in an emergency setting in Switzerland. The lower prevalence could be explained by the fact that the study involved multiple indications for diuretics, the emergency setting and the fact that most patients were on a single diuretic; by contrast, most of the patients in the index study were on multiple diuretics and it involved patients attending a routine clinic visit. It is also possible that patients in developing countries are less likely to be monitored properly for various reasons leading to a higher likelihood of developing hyperkalemia and hence a higher prevalence.

Factors which were significantly associated with hyperkalemia in this study were NYHA class III or IV, history of confusion, nausea, muscle weakness and vomiting, higher BP and history of renal failure. Similar factors have been reported by in multiple studies conducted elsewhere.^15,14,17, 18^

The prevalence of hypokalemia in this study was 4.3%, which is lower than the previously reported range of 19 to 54% among HF patients. This variation largely depends on the definition of hypokalemia and patient characteristics.^19^ It is important to note that the cited range is based on studies conducted before the extensive use of medications such as beta-blockers, ACE-inhibitors, ARB and MRAs, all of which increase serum potassium levels and thus counteract hypokalemia. Most of the patients in the current study were on these medications. In addition, our study population consisted of outpatients, in contrast to the hospitalised patients who tend to have higher levels of hypokalemia.^6,20,21^

Factors which were significantly associated with hypokalemia were high systolic and diastolic blood pressure. These factors are described for the first time specifically in patients with heart failure on diuretics.

## CONCLUSION

Hyperkalemia occured in 19% of adults with heart failure seen in the medical outpatient whereas hypokalemia affects 4.3%. Factors associated with potassium abnormalities include; medication use, kidney disease and more severe heart failure. Healthcare providers should ensure that all patients with these associated factors receive regular electrolyte testing. In addition, preventive strategies should be put into place to prevent electrolyte abnormalities in this population, including judicious prescription of medications and, if possible, avoidance of NSAIDs. Electrolytes should be part of the baseline investigation to all patients with heart failure starting treatment, and should be closely monitored in every clinic visit for early detection of these abnormalities and possible intervention, including dose adjustments.

### Study Limitations

This study faces several limitations. Firstly, due to its retrospective nature, information on some patients was incomplete, potentially introducing bias into our findings. Secondly, the sample originated from clinic attendees at a tertiary zonal health facility, whose characteristics might not accurately represent those of heart failure patients at lower levels of healthcare. Despite these limitations, the study offers valuable local data that could assist healthcare providers in managing patients with heart failure.
